# Colour map displays

**DOI:** 10.1186/s13244-025-01970-2

**Published:** 2025-06-17

**Authors:** Nandita M. deSouza, Miha Fuderer, Nico Sollmann, Barbara Daria Wichtmann, Xavier Golay

**Affiliations:** 1 The Institute of Cancer Research, London, UK; 2 The Royal Marsden NHS Foundation Trust, London, UK; 3 Radiotherapy, Division Imaging and Oncology, University Medical Center Utrecht, Utrecht, The Netherlands; 4 Department of Diagnostic and Interventional Radiology, University Hospital Ulm, Ulm, Germany; 5 Department of Diagnostic and Interventional Neuroradiology, School of Medicine, Klinikum rechts der Isar, Technical University of Munich, Munich, Germany; 6 Clinic of Neuroradiology, University Hospital Bonn, Bonn, Germany; 7 Queen Square Institute of Neurology, University College London, London, UK; 8 Gold Standard Phantoms, Sheffield, UK; 9 Bioxydyn, Manchester, UK

The use of colour-encoded images, displayed as colour maps, is becoming increasingly ubiquitous in radiology as quantitative data are exploited more routinely, both in research as well as clinical routine. Nevertheless, the schemes adopted in colour map displays are usually arbitrary and merely reflect individual preferences or default settings of their processing tool, rather than conforming to any standards set by international bodies. Results from schemes such as the rainbow or jet colour maps that are frequently used can therefore be misinterpreted, as the linearity of the colour scale is not evident, and the brightest colour (yellow) is not at the top of the scale. This is problematic, particularly when viewed by users who lack the expertise to appreciate the nuances in the underlying data, and can potentially lead to erroneous assumptions and therefore affect diagnosis accuracy, leading to the implementation of patient management strategies that may be inappropriate and unwarranted. An initial attempt at standardising colour displays of quantitative magnetic resonance imaging (MRI) was therefore undertaken by the Quantitative MR Group of the International Society of Magnetic Resonance in Medicine (ISMRM) to meet the universally recognised desirable features of colour maps [1, 2]:

1. Even colour contrast along the colour gradient for accurate representation of data (only a perceptually uniform colour map weights the same data variation equally across the data space and limits adding visual errors to the data).
2. High overall colour contrast of the colour gradient (ensuring good visibility of imaged features in the data for people with normal colour vision).
3. Monotonic and overall high lightness contrast of the colour gradient (ensuring universal and good visibility of imaged features in the data, also for people with impaired colour vision).
4. Intuitive and constant magnitude of the colour gradient along the whole colour scale (a perceptually ordered colour map does not break the correspondence between colour and numeric values and enables qualitative understanding of a dataset).
5. Recognisability, from the colour scheme, of the type of information that is presented (by fostering a uniform, coordinated use of individual colour schemes for given types of data, not only over many users, institutes, countries, and manufacturers, but also over time).

The Colour Recommendation Committee, which was launched from the Quantitative MR Study Group of the ISMRM, reviewed the limitations of colour maps as they are currently presented in the literature with regard to five quantitative biomarkers used routinely in clinical practice: relaxometry (T1, R1, T2, T2*, R2, and R2*), dynamic contrast-enhanced metrics (permeability (*K*^trans^), initial area under gadolinium curve (iAUGC), relative cerebral blood volume (rCBV), mean blood flow (MBF)), diffusion-related metrics (apparent diffusion coefficient (ADC), fractional anisotropy (FA), tractography), magnetic resonance elastography (MRE), and proton density fat fraction (PDFF) [3]. Current conformance with the desirable features of colour maps was found lacking in most cases. To initiate a process of standardisation, the Colour Recommendation Committee conducted a Delphi process to provide guidelines through consensus on the generation of colour maps [4]. This only addressed MR relaxometry in the first instance, as the requirements for quantitative biomarkers derived using different MRI techniques vary considerably. Recommendations were generated by a Delphi process consisting of four rounds, addressing a panel of 81 experts. Questions were devised by a multidisciplinary committee and included questions on whether the use of colour at all was warranted. The proposed colour maps were based on previous proposals [5], but modified for perceptual linearity and readability by colour-blind people. Questions devised by a multidisciplinary committee were circulated to the Quantitative MR Study Group of the ISMRM and European subspecialist society representatives. Respondents received feedback after each round to aid consensus. Responses on a 9-point Likert scale were summarised to “agree”, “neutral”, and “disagree” categories. A consensus of 75% was the threshold for items reaching recommendation. Of the 58 experts who responded to Round 1, 48 (45% medical, 47% physicists, 8% not specified) completed all four rounds. There was consensus that the logarithm-processed Lipari colour map for T1 and the logarithm-processed Navia colour map for T2 may be most suitable [4]. Colour bars were deemed mandatory, as was a specific value indicating “invalidity”. There was no consensus on whether to fix ranges by anatomy, and this will require further work that specifically focuses on range recommendations.

As a result of this Delphi-based colour map recommendations for MR relaxometry, there now exists an internationally agreed consensus recommendation for colour displays of MR relaxometry: the logarithm-processed Lipari colour map for displaying T1 and R1 values and the logarithm-processed Navia colour-map for displaying T2, R2, T2*, and R2* [4]. This standard is recommended for widespread adoption for use in scientific reports as well as in clinical practice and will hopefully spawn further international efforts on the choice of colour displays to be used for other quantitative MRI-derived biomarkers. Efforts in this direction are increasingly important as we embrace automation in radiology with the embedding of artificial intelligence into our routine outputs.
